# Transthyretin Promotes Axon Growth *via* Regulation of Microtubule Dynamics and Tubulin Acetylation

**DOI:** 10.3389/fcell.2021.747699

**Published:** 2021-11-08

**Authors:** Jessica Eira, Joana Magalhães, Nídia Macedo, Maria Elena Pero, Thomas Misgeld, Mónica M. Sousa, Francesca Bartolini, Márcia A. Liz

**Affiliations:** ^1^ICBAS, Instituto de Ciências Biomédicas Abel Salazar, Universidade do Porto, Porto, Portugal; ^2^Neurodegeneration Team, Nerve Regeneration Group, Instituto de Biologia Molecular e Celular-IBMC, and i3S – Instituto de Investigação e Inovação em Saúde, Universidade do Porto, Porto, Portugal; ^3^Department of Pathology & Cell Biology, Columbia University, New York, NY, United States; ^4^Department of Veterinary Medicine and Animal Production, University of Naples Federico II, Naples, Italy; ^5^Institute of Neuronal Cell Biology, Technical University of Munich, German Center for Neurodegenerative Diseases (DZNE), Munich Cluster of Systems Neurology (SyNergy), Munich, Germany; ^6^Nerve Regeneration Group, Instituto de Biologia Molecular e Celular-IBMC, and i3S – Instituto de Investigação e Inovação em Saúde, Universidade do Porto, Porto, Portugal

**Keywords:** transthyretin, nerve biology, axon growth, microtubules, tubulin acetylation, transthyretin amyloid polyneuropathy

## Abstract

Transthyretin (TTR), a plasma and cerebrospinal fluid protein, increases axon growth and organelle transport in sensory neurons. While neurons extend their axons, the microtubule (MT) cytoskeleton is crucial for the segregation of functional compartments and axonal outgrowth. Herein, we investigated whether TTR promotes axon elongation by modulating MT dynamics. We found that TTR KO mice have an intrinsic increase in dynamic MTs and reduced levels of acetylated α-tubulin in peripheral axons. In addition, they failed to modulate MT dynamics in response to sciatic nerve injury, leading to decreased regenerative capacity. Importantly, restoring acetylated α-tubulin levels of TTR KO dorsal root ganglia (DRG) neurons using an HDAC6 inhibitor is sufficient to completely revert defective MT dynamics and neurite outgrowth. In summary, our results reveal a new role for TTR in the modulation of MT dynamics by regulating α-tubulin acetylation *via* modulation of the acetylase ATAT1, and suggest that this activity underlies TTR neuritogenic function.

## Introduction

Transthyretin (TTR) is a homotetrameric protein predominantly synthesized in the liver and the choroid plexus of the brain that functions physiologically as a thyroxin (Woeber and Ingbar, 1968) and retinol transporter (Goodman, 1984) in the plasma and cerebrospinal fluid. When mutated, TTR aggregates and deposits in the peripheral nervous system (PNS), leading to a distal axonopathy – Transthyretin Amyloid Polyneuropathy (ATTR-PN; Plante-Bordeneuve and Said, 2011). The initial clinical manifestations in ATTR-PN patients point to an early sensory nerve degeneration resulting in abnormal pain sensation. Interestingly, TTR KO mice display sensorimotor impairment and decreased neurite outgrowth and axonal transport in sensory dorsal root ganglia (DRG) neurons (Fleming et al., 2007; Fleming et al., 2009), suggesting that wild type (WT) TTR participates in the physiology of sensory axons. However, the cellular and molecular details underlying the physiological role of TTR on peripheral nerve biology remain to be deciphered.

Microtubules (MTs) play a fundamental role in neuronal health by providing structural support and establishing the tracks for axonal transport. In addition, modulation of MT dynamics, which reflects the change between extensive phases of MT growth followed by rapid disassembly and regrowth at the MT growing ends, is essential for proper axon growth and synaptic function during development or nerve injury (Murillo and Mendes Sousa, 2018; Parato and Bartolini, 2021; Pinto-Costa and Sousa, 2021; Waites et al., 2021). After injury, peripheral axons form a growth cone invaded by dynamic MTs, while axonal shafts contain more stable MT bundles (Erturk et al., 2007). In addition to the action of MT-associated proteins (MAPs), tubulin isoforms and nucleotide state, tubulin post-translational modifications (PTMs) are important regulators of MT dynamics (Moutin et al., 2020). Among them, α-tubulin acetylation, a tubulin PTM associated with stable MTs, plays an essential role in maintaining touch sensitivity in mechanosensory neurons (Morley et al., 2016; Yan et al., 2018). Furthermore, increasing acetylated α-tubulin by inhibition of the enzyme responsible for deacetylation, HDAC6, has proved to be a successful therapeutic approach in toxic and inherited cases of peripheral neuropathy (Krukowski et al., 2017; Benoy et al., 2018; Prior et al., 2018), and in axon regeneration by increasing MT stability in the axonal shaft (Rivieccio et al., 2009).

In this study, we investigated whether TTR impacts the growth of peripheral axons *via* regulation of MT dynamics. We found that TTR increases MT dynamics in the distal end of growing axons while it stabilizes MT dynamics in the axonal shaft. We further show that TTR modulates axonal MT stability by regulating the levels of the tubulin acetylase α-tubulin acetyltransferase 1 (ATAT1), a function underlying its role as an axon growth promoter.

## Materials and Methods

### Animals

Mice were handled according to European Union and National rules. WT and TTR KO (Episkopou et al., 1993) littermates (in the Sv/129 background), were obtained from the offspring of heterozygous breeding pairs. Thy1.EB3-YFP (line J045), already successfully used to measure MT dynamics *in vivo* and *ex vivo* in axons of the peripheral and central nervous systems (Kleele et al., 2014; Sorbara et al., 2014), were crossed with TTR KO mice. The line J045 used in this work was shown to be a low EB3-YFP expressing line, being largely non-toxic and inert as previous established *in vitro* in classical transfection-based assays (Kleele et al., 2014). The resultant TTR KO(±).Thy1.EB3-YFP were intercrossed generating WT.Thy1.EB3-YFP and TTR KO.Thy1.EB3-YFP. All animals were maintained under a 12 h light/dark cycle and fed with regular rodent’s chow and tap water *ad libitum*. Genotypes were determined from ear extracted genomic DNA.

### Recombinant Transthyretin Production and Purification

Recombinant WT TTR was produced in *Escherichia coli* BL21(DE3) cells transformed with a pETF1 vector carrying human WT TTR (Goldsteins et al., 1997). We have chosen to use *E. coli* for TTR production, rather that human hepatocyte cell lines, to be able to carry experiments at a TTR concentration closer to what is found in the human plasma, approximately 300 μg/mL, an amount that could not be easily obtained from hepatocyte cell lines. TTR protein was isolated and purified as previously described (Silva et al., 2017). For cellular assays, recombinant TTR was detoxified using a high-capacity endotoxin removal resin (Thermo Scientific) and quantified using the Lowry based DC Protein Assay (Bio-Rad Laboratories).

### Primary Dorsal Root Ganglia Neuronal Cultures and Cell Treatment

Primary cultures of DRG neurons from 4- to 8-weeks-old mice were performed as described in Liz et al. (2014). For neurite outgrowth experiments with TTR addition, WT DRG neurons were plated in 20 μg/mL PLL + 5 μg/mL laminin coated 24 well plates at a density of 5,000 cells per well in complete medium [DMEM/F12 (Sigma-Aldrich, D8437) supplemented with 1 × B27 (Gibco), 1% penicillin/streptomycin (Gibco), 2 mM L-glutamine (Gibco), and 50 ng/mL NGF (Millipore, 01-125)] at 37°C and 5% CO_2_. 4 h after plating, DRG were treated with recombinant WT TTR (equal volume of PBS for the control condition) at a concentration of 300 μg/mL and incubated for 12 h at 37°C and 5% CO_2_. For EB3-GFP transfection experiments, the 4D Nucleofector Amaxa system (Lonza, Barcelona, Spain, CM#137 program) was used and WT DRG neurons were nucleofected at a density of at least 200,000 cells/condition with a truncated version of EB3-GFP (a construct containing amino acids 1–200 of EB3, artificially dimerized by the addition of the leucine zipper domain of GCN4, cloned into the pEGFP-N1 vector, that efficiently accumulates at MT tips) (Komarova et al., 2009). After transfection, cells were left in suspension for 24 h and then plated on 20 μg/mL PLL + 5 μg/mL laminin coated 35 mm μ-dishes (ibidi) at a density of 15,000 neurons per dish in phenol-free DMEM/F12 supplemented as above. 4 h after plating, DRG neurons were treated with recombinant WT TTR at a concentration of 300 μg/mL and incubated for 12 h at 37°C and 5% CO_2_. To assess the impact of HDAC6 inhibition, using ACY-738 (provided by Acetylon Pharmaceuticals) (Jochems et al., 2014), or of TTR addition on acetylated α-tubulin levels and MT dynamics, WT.Thy1.EB3-YFP and TTR KO.Thy1.EB3-YFP DRG neurons were used. Neurons were plated on 20 μg/mL PLL + 5 μg/mL laminin coated 24 well plates at a density of 5,000 cells per well for acetylated α-tubulin and βIII-tubulin staining, or in 8 well μ-dishes (ibidi) at a density of 8,000 cells per well for EB3 live imaging of MT dynamics, using DMEM/F12 (Sigma-Aldrich, D8437) supplemented with 1 × B27 (Gibco), 1% penicillin/streptomycin (Gibco), 2 mM L-glutamine (Gibco), 50 ng/mL NGF (Millipore, 01-125), 60 μM 5-Fluoro-2′-deoxyuridine (FluoU) and 100 nM of ACY-738 in DMSO (equal volume of DMSO was used for the control condition), and incubated at 37°C and 5% CO_2_. At DIV3 half of the medium was changed by new medium supplemented with twice the concentration of ACY-738 and FluoU. At DIV4, 2 h before fixing, medium was supplemented with ACY-738. In the case of TTR addition, the protein was added at 300 μg/mL at DIV1 and at DIV3. For live imaging experiments, 2 h before imaging, the medium was changed to phenol-free DMEM/F12 supplemented as above; imaging was started in the ACY-738 treated conditions to minimize the effect of its short lifetime (Jochems et al., 2014). For neurite outgrowth assessment with HDAC6 inhibition using ACY-738, 4 to 8-weeks-old WT and TTR KO DRG neurons were plated on 20 μg/mL PLL + 5 μg/mL laminin coated 24 well plates at a density of 5,000 cells per well in complete medium supplemented with 100 nM of ACY-738 in DMSO (equal volume of DMSO was used for the control condition) at 37°C and 5% CO_2_ for 16 h.

### Sciatic Nerve Crush

12-weeks-old mice were anesthetized with isoflurane and a 4-mm-long incision was made in the shaved thigh skin. For nerve crush, the sciatic nerve was exposed and crush was performed using Pean forceps, three times during 10 s. To standardize the procedure, the crush site was maintained constant for each animal at 5 mm distally to the notch. A single skin suture, immediately above the crush site, served as an additional reference. After surgery, animals were allowed to recover for 3 days after which mice were sacrificed in a CO_2_ chamber and the sciatic nerves (ipsilateral i.e., crushed- SNC) and the contralateral (uninjured- uninj) were subsequently collected for either immunohistochemistry or live cell imaging.

### EB3 Live Imaging for the Analysis of Microtubule Dynamics

For analysis of MT dynamics, DRG neurons were recorded for 2 min (60 frames total) in phenol-free DMEM/F12 supplemented as mentioned above, at 37°C and 5% CO_2_, using a Spinning Disk Confocal System Andor Revolution XD with an iXonEM + DU-897 camera and an IQ 1.10.1 software (ANDOR Technology). For the *ex vivo* imaging, sciatic nerves from WT.Thy1.EB3-YFP and TTR KO.Thy1.EB3-YFP were collected 3 days post injury and placed in 35 mm μ-Dish (ibidi) with phenol-free DMEM/F12, and recordings were performed as described above. For the quantification of the different EB3 dynamics parameters, kymographs were made using the Fiji KymoResliceWide plugin (distance, *x* axis; time, *y* axis). Start and end positions of the traces were defined using the Fiji Cell Counter plugin generating comet length (i.e., comet movement length in micrometers), comet duration (i.e., comet lifetime in seconds), and growth rate (comet length/comet duration). Comet density in the growth cone was calculated as the number of comets in 30 consecutive frames divided by the number of quantified frames and growth cone area. Comet density in the axonal shaft was determined as the number of comets per axon area per minute.

### Analysis of Sciatic Nerve Ultrastructure and Microtubule Density

For ultrastructure analysis of MT density, 12-weeks-old WT and TTR KO mice were sacrificed using a CO_2_ chamber and sciatic nerves were collected and fixed in 4% glutaraldehyde in 0.1 M sodium cacodylate buffer (pH 7.4) for 5 days and processed for ultrathin sections as previously described (da Silva et al., 2014). For MT density analysis, 30–60 axons of 2–4 μm of diameter were analyzed using Photoshop CS3 for image processing and mounting.

### Immunoblotting

Protein lysates from 12-weeks-old quick frozen sciatic nerves from WT and TTR KO mice, or from WT or TTR KO DIV4 DRG neuronal cultures, were prepared in ice-cold RIPA lysis buffer (1% Triton X-100, 0.1% SDS, 140 mM NaCl, 1x TE pH 8, 1x protease inhibitor cocktail (Merck) and 1 mM sodium orthovanadate), sonicated (2 × 10 cycles, output power 50 Watts, Branson sonifier 250) and cleared by centrifugation at 15,000 rpm for 10 min at 4^*o*^C. 20 μg of protein extracts for analysis of MT severing enzymes or 2.5 μg of protein extracts for analysis of tubulin proteins were separated under denaturing conditions and transferred to Amersham Protran Premium 0.45 μm nitrocellulose membranes (GE Healthcare Life Sciences) prior to blocking in 5% non-fat dried milk in TBS-T for 1 h at room temperature. Membranes were probed overnight at 4^*o*^C with the following primary antibodies (in 5% BSA in TBS-T): rabbit anti-katanin (1:500, Proteintech, 17560-1-AP), mouse anti-spastin (1:500, Santa Cruz Biotechnology, sc-81624), mouse anti-βIII-tubulin (1:10,000; Promega, G7121), mouse anti-α-tubulin (0.5 μg/mL; DSHB, 12G10), rabbit anti-ATAT1 (1:500; Novus Biological NBP1-57690), rabbit anti-HDAC6 (1:1,000; Cell Signaling, D21B10), mouse anti-acetyl α-tubulin (1:40,000; Sigma-Aldrich, T7451), rabbit anti-ERK (1:1,000, Cell Signaling, 9102), mouse anti-GAPDH (1:1,000; Santa Cruz Biotechnology, sc-166574) and rabbit anti-vinculin (3:10,000; ThermoFisher Scientific, 700062). Secondary antibodies were used in 5% non-fat dried milk in TBS-T for 1 h at room temperature. Secondary antibodies were mouse IgGκ light chain conjugated with horseradish peroxidase (HRP) (1:2,000; Santa Cruz Biotechnology, sc-516102) and goat anti–rabbit IgG conjugated with HRP (1:10,000; Jackson ImmunoResearch Labs, 111-035-003). Immunodetection was performed by chemiluminescence using ECL (Millipore, WBLUR0500) and quantified using ImageJ software. Being acetylated α-tubulin a major focus of this work, we performed an experiment for the validation of our batch of monoclonal anti-acetylated α-tubulin antibody, highly used in the literature to detect this tubulin PTM (Kalebic et al., 2013). We treated WT DRG neurons with 2.5 μM ACY-738 (HDAC6 inhibitor) (Supplementary Figure 1) and recapitulated the conditions to increase the levels of acetylated α-tubulin without altering total α-tubulin levels in neurons (Benoy et al., 2017).

### Immunohistochemistry

Sciatic nerves from 12-weeks-old WT and TTR KO mice were perfused with PBS for 5 min followed by 4% paraformaldehyde (PFA, pH 7.4) in PBS (40 ml). Sciatic nerves were collected and maintained in 4% PFA for 24 h and then cryopreserved in 30% sucrose for 48 h. Cryopreserved sciatic nerves were embedded in Optimum Cutting Temperature (OCT) compound (ThermoFisher Scientific), frozen and cut longitudinally (Cryostat Leica CM3050S) in 12 μm thick sections. For SCG-10 staining, sections were blocked with 5% fetal bovine serum (FBS), 0,1% TritonX-100 in PBS for 1 h at room temperature, followed by incubation with the primary antibody rabbit anti-SCG-10 (1:5,000, Novus Biological, NBP1-49461) diluted in blocking buffer. Sections were then incubated with donkey anti-rabbit Affinipure Alexa Fluor 594 (1:1,500, Jackson ImmunoResearch, 711-605-152) diluted in blocking buffer for 2 h at RT. Then sections were washed with 0.05% Triton X-100 in PBS and mounted with ibidi mounting medium with dapi (ibidi, 50001). For acetylated α-tubulin and βIII-tubulin staining, sections were blocked with 5% normal donkey serum (NDS) containing 0,3% Triton in PBS for 1 h at room temperature, followed by incubation for two overnights at 4^*o*^C with mouse anti-acetylated α-tubulin (1:500, Sigma-Aldrich, T7451) and rabbit anti-βIII-tubulin (1:500, Abcam, 1967-1) diluted in blocking buffer. Sections were then incubated with donkey anti-mouse Alexa Fluor 568 (1:1,000, Alfagene, A10037) and donkey anti-rabbit Alexa Fluor 647 (1:500, Jackson ImmunoResearch, 711-605-152) diluted in blocking buffer for 1 h at room temperature, washed in PBS and mounted in ibidi mounting medium (ibidi, 50001).

### Immunocytochemistry

For neurite outgrowth experiments, neurons were fixed with 4% PFA, blocked with 5% NDS containing 0,4% Tween for 1 h at room temperature, incubated with mouse anti–βIII-tubulin (1:2,000; Promega, G7121) overnight at 4°C followed by incubation with secondary antibody (donkey anti-mouse Alexa-Fluor 488, 1:1,000 Alfagene, A21202) for 1 h at room temperature. For acetylated α-tubulin and βIII-tubulin staining, DRG neurons, were fixed with cytoskeleton preservation PHEM fixative (4% PFA, 4% sucrose, 0.25% glutaraldehyde, 0.1% Triton X-100, 300 mM PIPES, 125 mM HEPES, 50 mM EGTA and 10 mM magnesium chloride), permeabilized with 0.2% Triton X-100 for 5 min, quenched with 50 mM NH_4_Cl for 5 min and blocked with 2% FBS, 2% BSA and 0.2% fish gelatin in PBS for 1 h at room temperature. Incubation with mouse anti-acetylated α-tubulin (1:5,000; Sigma-Aldrich, T7451) and rabbit anti-βIII-tubulin (1:500, Abcam, 1967-1) was performed in 10% blocking buffer overnight at 4°C. Incubation of the secondary antibodies donkey anti-mouse Alexa Fluor 568 (1:1,000, Alfagene, A10037) and donkey anti-rabbit Alexa Fluor 647 (1:500, Jackson ImmunoResearch, 711-605-152) was performed in 10% blocking buffer for 1 h at room temperature. Cells were mounted in Fluoromount (Southern Biotech).

### Imaging and Quantification

For neurite outgrowth experiments, images were acquired either using an epifluorescence microscope Zeiss AxioImager Z1 with an Axiocam MR3.0 camera and Axiovision 4.7 software or a Leica DMI6000 FFW microscope with an HC PL Fluotar 10x objective and a LAS X software operating the navigator feature. The longest neurite tracing analysis was performed using Fiji software and neuronJ plugin. For analysis of axonal regeneration with SCG-10 staining, sections were imaged with the IN Cell Analyzer 2000 Bioimager (GE Heathcare). Quantification of SCG-10 fluorescence was performed using Fiji in longitudinal sections, by plotting the mean gray values in relation to the distance of the lesion epicenter (LE). The intensity was normalized to the intensity at the LE and presented as the regeneration index. Axonal regeneration was additionally quantified distally to the injury site by measuring the distance from the distal tip of the regenerating axons to the lesion border (a minimum of four sections per animal were analyzed). To define the lesion area, DAPI counterstaining was used to detect the accumulation of nuclei of inflammatory cells within the crush site. For acetylated α-tubulin/βIII-tubulin quantifications in sciatic nerves, WT and TTR KO sections were imaged in a laser scanning confocal microscope Leica TCS SP8, using the PL APO 10× objective. For acetylated α-tubulin/βIII-tubulin quantifications in DRG neurons, Leica DMI6000 FFW microscope with an HCX PL Fluotar 20× objective was used with LAS X software. Ratiometric analysis of acetylated α-tubulin/βIII-tubulin in sciatic nerve sections and DRG neurons was performed by determination of regions of interest selecting stretches of βIII-tubulin + axons, randomly, and using ImageJ software.

### Statistics

All statistical tests were performed using GraphPad Prism 7 software. Unless otherwise stated, the Student’s *t*-test or the One-way ANOVA with Tukey’s multiple comparisons test were used. Statistical tests and sample sizes are indicated in figure legends and significance was defined as ^∗^*p* < 0.05; ^∗∗^*p* < 0.01; ^∗∗∗^*p* < 0.001; and ^****^p < 0.0001; ns, not significant.

## Results and Discussion

### Transthyretin Modulates Microtubule Dynamics in Cultured Dorsal Root Ganglia Neurons to Promote Neurite Outgrowth

**FIGURE 1:**
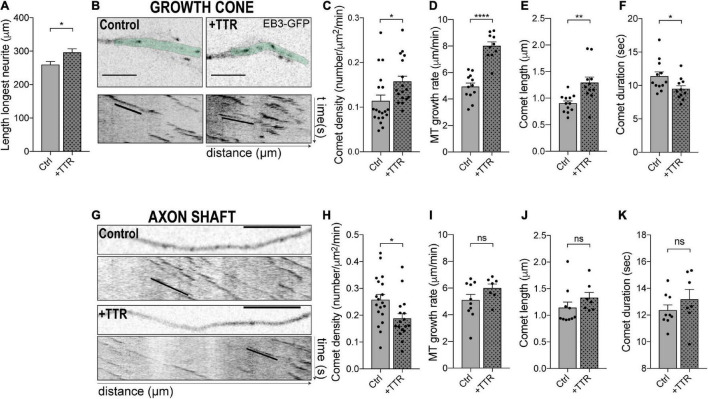
Transthyretin (TTR) regulates microtubule (MT) dynamics in dorsal root ganglia (DRG) neurons. **(A)** Quantification of the length of the longest neurite in DRG neurons either untreated (Ctrl) or treated with soluble wild type (WT) TTR (+TTR). Data represent mean ± SEM (*n* = 112–197 neurons/condition; representative of three independent experiments). Statistical significance determined by Student’s *t*-test: *P < 0.05. **(B)** Representative still images of the growth cones from DRG neurons transfected with EB3-GFP either untreated (Control) or treated with soluble WT TTR (+TTR) (top) and kymographs from the growth cone region highlighted in green (bottom). **(C–F)** Quantifications of different MT dynamics parameters in the growth cone including comet density **(C)**, MT growth rate **(D)**, comet length **(E)**, and comet duration **(F)**, related to panel **(B)**. Results are plotted as mean ± SEM (*n* = 10–19 growth cones/condition, representative of three independent experiments). **(G)** Representative still images of the neurite shafts from DRG neurons transfected with EB3-GFP either untreated (Control) or treated with WT soluble TTR (+TTR) and corresponding kymographs. **(H–K)** Quantifications of different MT dynamics parameters in the axon shaft including EB3 comet density **(H)**, MT growth rate **(I)**, comet length **(J)**, and comet duration **(K)** related to panel **(G)**. Results are plotted as mean ± SEM (*n* = 7–19 neurite shafts/condition, representative of three independent experiments). Statistical significance was determined by Student’s *t*-test: *P < 0.05; **P < 0.01; and ****P < 0.0001. ns, not significant. Scale bars: 5 μm.

TTR increases neurite outgrowth of DRG neurons through an unknown mechanism (Fleming et al., 2007). Herein, we determined whether TTR neuritogenic activity is mediated by its interference with MT dynamics. WT TTR promoted neurite outgrowth in primary cultures of dissociated DRG neurons as determined by a significant increase in the length of the longest neurite (Figure 1A). We tested whether this effect correlated with the modulation of MT dynamics and found that in the growth cone of DRG neurons, transfected with the MT plus-tip binding protein EB3, WT TTR significantly increased the density of dynamic EB3 comets (Figures 1B,C). Additionally, TTR extended EB3 growth rate and comet growth length, while it decreased duration of growth (Figures 1B,D–F and Supplementary Videos 1, 2). These results suggest that TTR promotes neurite outgrowth by increasing MT plus end dynamics in the growth cone of DRG neurons.

MT dynamics is highly dependent on subcellular localization. This is especially relevant in neurons, where MTs are not attached to the centrosome and MT plus ends are subjected to different stimuli depending upon whether their location is proximal or distal relatively to the cell body. In addition, while in the growth cone MTs must be highly dynamic to increase the association of dynein with + TIP proteins to promote outgrowth (McElmurry et al., 2020) and respond to extracellular stimuli during neurite extension, stable MTs are needed in the axonal shaft to drive axon outgrowth (Erturk et al., 2007; Bradke et al., 2012; Murillo and Mendes Sousa, 2018) and increase axonal transport to promote regeneration (Reed et al., 2006). The density of dynamic MT ends serves as a general marker of MT dynamics (Kleele et al., 2014). We investigated the effect of WT TTR on the dynamic state of MTs in the neurite shaft of DRG neurons and found that, in contrast to what was observed in the growth cone, it decreased EB3 comet density (Figures 1G,H), while it had no impact on comet growth rate, comet length and growth duration (Figures 1G,I–K). These data demonstrate a novel function for TTR in regulating MT dynamics and offer a mechanistic explanation of the pro-growth activity of TTR in axon extension by differential regulation of MT dynamics at the growth cone and in the axonal shaft.

### Transthyretin KO Sciatic Nerve Axons Fail to Increase Microtubule Dynamics and to Regenerate Their Axons in Response to Injury

**FIGURE 2:**
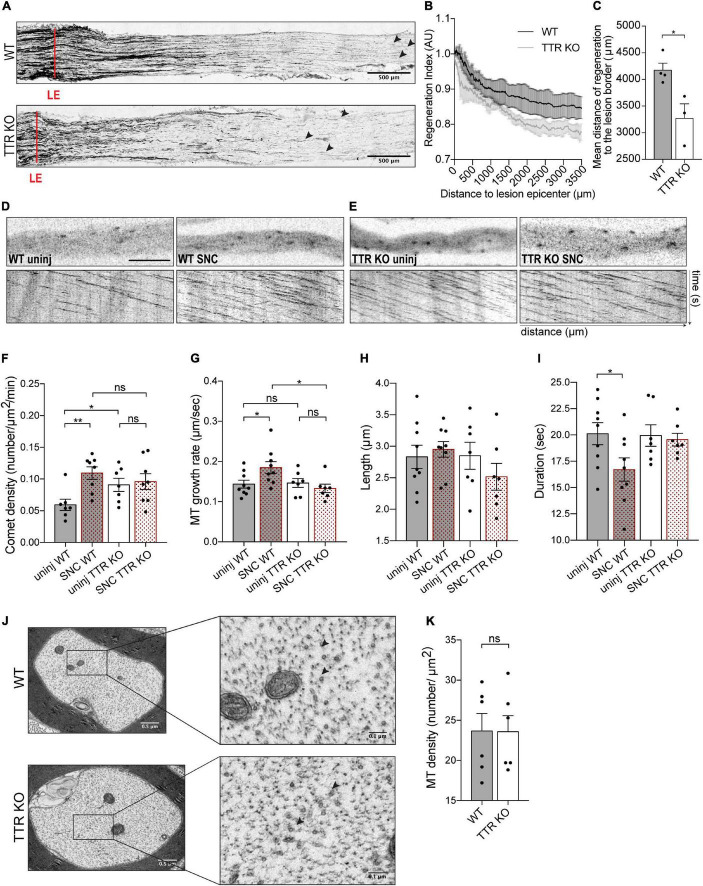
Sciatic nerve axons from TTR KO mice have higher dynamic MT density but fail to increase MT dynamics and to regenerate their axons in response to sciatic nerve crush. **(A)** SCG-10 staining of longitudinal sciatic nerve sections from WT and TTR KO mice at 3 days postinjury; red lines indicate the lesion epicenter (LE), arrowheads highlight the longest regenerating axons. Scale bars: 500 μm. **(B)** Regeneration index vs. distance to LE. **(C)** Mean distance of SCG-10 + sciatic nerve axons regenerating distally to the lesion edge. Data represent mean ± SEM (*n* = 4 animals/genotype). *P < 0.05 by Student’s *t*-test. **(D)** Representative still images of the axonal shaft from a WT-Thy1-EB3-YFP uninjured nerve (WT uninj) with correspondent kymograph and of an axonal region distal to the lesion site from a WT-Thy1-EB3-YFP crushed nerve (WT SNC) with correspondent kymograph. Scale bar: 5 μm. **(E)** Representative still images of the axonal shaft from a TTR KO-Thy1-EB3-YFP uninjured nerve (TTR KO uninj) with correspondent kymograph and of an axonal region distal to the lesion site from a TTR KO-Thy1-EB3-YFP crushed nerve (TTR KO SNC) with correspondent kymograph. **(F–I)** Quantifications of MT dynamics parameters including EB3 comet density **(F)**, MT growth rate **(G)**, comet length **(H)**, and comet duration **(I)** from WT-Thy1-EB3-YFP (WT) and TTR KO-Thy1-EB3-YFP (TTR KO) nerves either uninjured (uninj) or crushed (SNC). Results are plotted as mean ± SEM (*n* = 7–9 animals/condition, 6–12 axons/animal). *P < 0.05, **P < 0.01. **(J)** Representative electron microscopy images of individual axons from WT and TTRKO sciatic nerves and respective high magnification representations. MTs are represented by black arrowheads. **(K)** Quantification of axonal MT density from WT and TTR KO sciatic nerves. Results are plotted as mean ± SEM (*n* = 6 animals/genotype, 30–60 axons/animal). Statistical significance determined by Student’s *t*-test: ns, not significant.

Based on the observation that TTR modulates MT dynamics *in vitro*, we investigated whether genetic ablation of TTR affects regenerative growth and MT dynamics after sciatic nerve injury *in vivo*. To evaluate the role of TTR in axonal regeneration *in vivo*, we used an experimental model of sciatic nerve crush in 12-weeks-old TTR KO mice. The mouse sciatic nerves were injured, and 3 days after, axon regeneration was assessed in the crushed nerves by SCG-10 immunoreactivity. We observed that TTR KO mice were less efficient in regenerating their axons as their regeneration index, measured over 3 mm away from the injury site, was significantly decreased when compared to WT control littermates (Figures 2A,B). Accordingly, the mean distance of SCG-10 positive regenerative axons from the lesion border was decreased in TTR KO mice (Figure 2C). These results confirm that TTR KO mice display decreased axon growth after injury.

To correlate these data with MT dynamics we used TTR KO-Thy1-EB3-YFP and their control WT-Thy1-EB3-YFP littermates. Following the same injury paradigm, at the 3rd day post-sciatic nerve crush we collected both the ipsilateral (crushed) and the contralateral (uninjured) nerves and performed *ex vivo* live imaging of EB3 comets. After crush, WT sciatic nerves mounted a response to injury characterized by increased comet density and growth rate, with decreased duration of growth, in growing axons distally to the crush site, when compared with uninjured nerves (Figures 2D,F–I and Supplementary Videos 3, 4). In contrast, injury to TTR KO mice, did not elicit an increase in comet density or speed, indicating a failure of the MT cytoskeleton to respond to nerve injury in the absence of TTR expression (Figures 2E–G and Supplementary Videos 5, 6). While analyzing uninjured sciatic nerves, we further noticed that, under naïve conditions, TTR KO mice had higher EB3 comet density in axonal shafts when compared to WT animals (Figures 2D–F). This observation is in agreement with the results observed *in vitro*, when comparing EB3 density in neurite shafts from DRG neurons untreated or treated with TTR (Figure 1G). Our *ex vivo* results suggest that loss of TTR promotes either MT nucleation or dynamics by inducing MT plus end rescue events in naïve axonal shafts. To clarify this, we assessed the density of MTs by electron microscopy of cross sections of WT and TTR KO uninjured sciatic nerves. No differences were scored in MT density between WT and KO axons (Figures 2J,K), indicating that in the absence of TTR, naïve axons in peripheral nerves intrinsically display more dynamic MT plus ends. In the axonal shaft, a tight balance between MT stability and dynamics is critical to enable normal axon physiology, in particular to sustain axonal transport (Murillo and Mendes Sousa, 2018). The increased MT dynamics in the shaft of TTR KOs may therefore underlie the dysregulation of such crucial balance and impact the capacity of axon regrowth post-injury.

### Transthyretin KO Mice Have Decreased Levels of α-Tubulin Acetylation in the Axons of Sciatic Nerves

**FIGURE 3:**
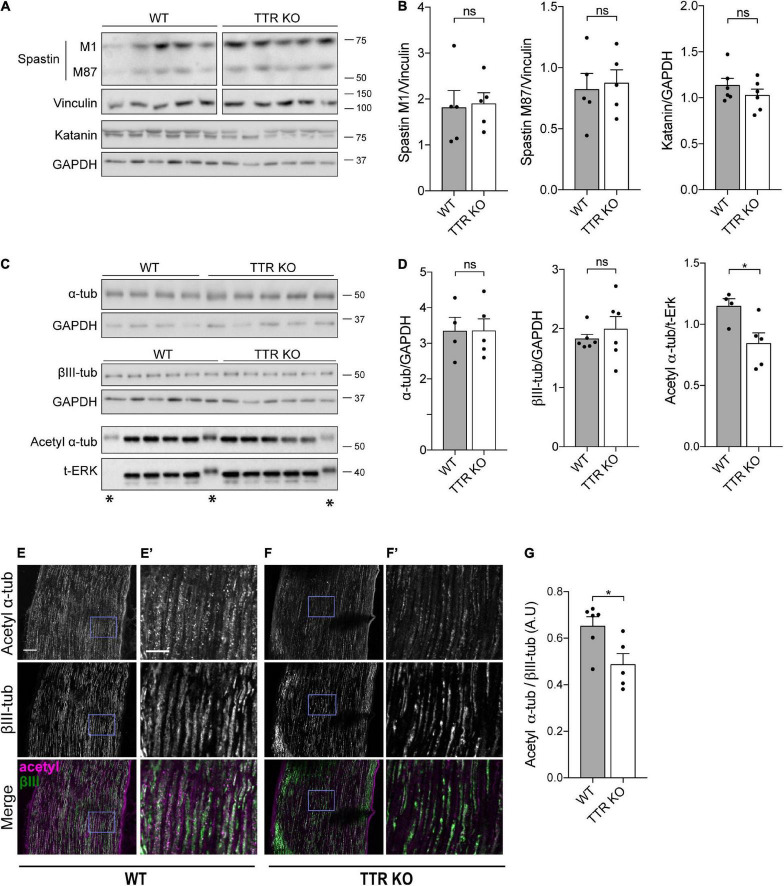
Transthyretin KO sciatic nerve axons have decreased levels of α-tubulin acetylation. **(A,B)** Western blot analysis **(A)** and respective quantification **(B)** showing MT severing enzyme levels in sciatic nerves of 12-weeks-old WT and TTR KO mice. Vinculin or GAPDH were used as loading controls. Data represent mean ± SEM (*n* = 5–6 animals/condition). ns – not significant by Student’s *t*-test. **(C,D)** Western blot analysis **(C)** and respective quantification **(D)** showing α-tubulin, βIII-tubulin, and acetyl α-tubulin levels in sciatic nerves of 12-weeks-old WT and TTR KO mice. GAPDH or ERK were used as loading controls. *Bands presenting an altered running pattern were not quantified. Data represent mean ± SEM (*n* = 4–6 animals/condition). Statistical significance determined by Student’s *t*-test: *P < 0.05. ns, not significant. **(E,F)** Representative images of 12-weeks-old WT (E) and TTR KO **(F)** sciatic nerves immunostained for acetylated α-tubulin and βIII-tubulin. Scale bar: 50 μm. **(E’,F’)** Zoomed in regions from panels **(E,F)**, respectively. Scale bar: 20 μm. **(G)** Quantification of the relative values of acetylated α-tubulin over βIII-tubulin. Data represent mean ± SEM (*n* = 5–6 animals/genotype, 45–57 axons/animal). Statistical significance was determined by Student’s *t*-test: *P < 0.05.

To investigate the molecular mechanisms underlying axonal MT instability in peripheral nerve axons of TTR KO mice, we determined whether this was related to differences in the levels of MT severing enzymes (Zhang et al., 2013). Western blot analysis showed no difference in the levels of both spastin and katanin in protein extracts from TTR KO and WT naïve sciatic nerves (Figures 3A,B). We analyzed the total levels of α- and βIII-tubulins and found no difference between WT and KO nerves (Figures 3C,D), a result in agreement with the observed similar MT density scored between the two genotypes by electron microscopy.

Acetylated α-tubulin accumulates onto previously stabilized MTs but its presence has been further associated to an increase in MT longevity given the enhanced ability of acetylated MTs to resist breakage (Xu et al., 2017). In addition, recent studies have shown that loss of acetylated α-tubulin leads to increased axonal MT debundling and MT plus-end dynamics with consequences in CNS development (Dan et al., 2018). Given the impact of acetylated α-tubulin levels on axonal integrity and sensory neuron function, we analyzed this tubulin PTM and found decreased levels of acetylated α-tubulin in protein extracts from TTR KO sciatic nerves by Western blot analysis (Figures 3C,D). Immunohistochemistry analysis of labeled axons confirmed that the ratio of acetylated α-tubulin vs. βIII-tubulin intensity was decreased in the sciatic nerves of TTR KO mice compared to their WT littermates (Figures 3E–G). These results strongly support the notion that loss of acetylated α-tubulin in TTR KO mice contributes to the inability of their nerves to regenerate and suggest that the intrinsic increase in MT dynamicity observed in the shaft may be a consequence rather than a cause of tubulin hypoacetylation.

### Transthyretin Promotes Neurite Outgrowth Through Modulation of Tubulin Acetylation by Regulating Acetylase α-Tubulin Acetyltransferase 1 Levels

**FIGURE 4:**
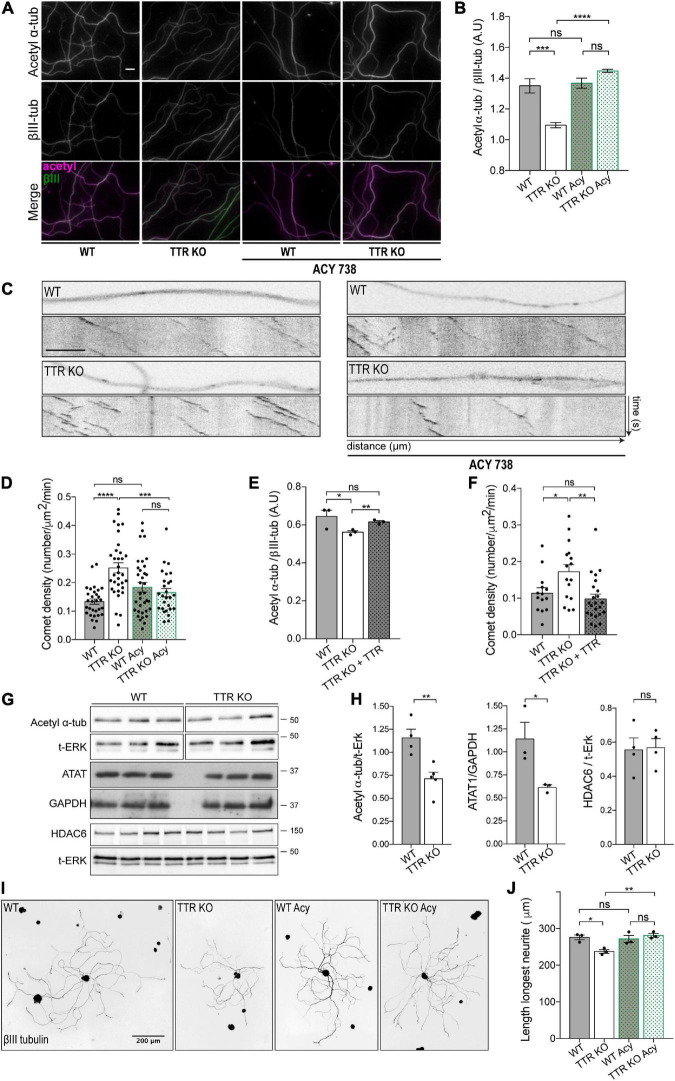
Transthyretin-mediated regulation of MT dynamics and axonal growth occurs *via* modulation of α-tubulin acetylation. **(A)** Representative images of acetylated α-tubulin (magenta) and βIII-tubulin (green) immunostaining of WT and TTR KO 4DIV DRG cultures untreated and treated with ACY-738. Scale bar: 10 μm. **(B)** Quantification of the relative values of acetylated α-tubulin over βIII-tubulin. Data represent mean ± SEM (*n* = 4 independent samples/condition; 50 axons/sample. Statistical significance determined by One-way ANOVA with Tukey’s multiple comparisons test: ***P < 0.001; ****P < 0.0001, ns, not significant. **(C)** Representative still images of the axonal shafts from DIV4 WT-Thy1-EB3-YFP (WT) and TTR KO-Thy1-EB3-YFP (TTR KO) DRG neurons either untreated or treated with ACY-738 and correspondent kymographs. Scale bar: 5 μm. **(D)** Quantification of comet density in neurite shafts from DIV4 WT-Thy1-EB3-YFP and TTR KO-Thy1-EB3-YFP DRG neurons untreated (WT; TTR KO) or treated with ACY-738 (WT Acy; TTR KO Acy). Results are plotted as mean ± SEM (23–33 neurite shafts/condition; representative experiment). Statistical significance determined by One-way ANOVA with Tukey’s multiple comparisons test: ***P < 0.001; ****P < 0.0001, ns, not significant. **(E)** Quantification of the relative values of acetylated α-tubulin over βIII-tubulin in neurite shafts from DIV4 WT and TTR KO untreated (TTR KO) or treated with soluble WT TTR (TTR KO + TTR). Data represent mean ± SEM (*n* = 3 independent experiments. 50 axons/condition/experiment). Statistical significance determined by Student’s *t*-test: *P < 0.05; **P < 0.01. ns, not significant. **(F)** Quantification of comet density in neurite shafts from DIV4 WT-Thy1-EB3-YFP (WT) and TTR KO-Thy1-EB3-YFP untreated (TTR KO) or treated with soluble WT TTR (TTR KO + TTR). Results are plotted as mean ± SEM (14–23 neurite shafts/condition; representative experiment). Statistical significance determined by One-way ANOVA with Tukey’s multiple comparisons test: *P < 0.05; **P < 0.01, ns, not significant. **(G,H)** Representative western blot **(G)** and respective quantification **(H)** showing acetyl α-tubulin, HDAC6 and ATAT1 levels in protein extracts from DIV4 WT and TTR KO neurons. ERK or GAPDH were used as a loading control. Data represent mean ± SEM (*n* = 3–4 independent experiments). *P < 0.05; **P < 0.01 by Student’s *t*-test. ns, not significant. **(I)** Representative anti-βIII-tubulin immunofluorescence images of DIV1 WT and TTR KO DRG neurons untreated (WT; TTR KO) or treated with ACY-738 (WT Acy; TTR KO Acy). **(J)** Data represent mean ± SEM (*n* = 3 independent experiments. 50–114 neurons/condition/experiment). Statistical significance determined by One-way ANOVA with Tukey’s multiple comparisons test: *P < 0.05; **P < 0.01, ns, not significant.

To investigate whether loss of acetylated α-tubulin in TTR KO axons underlies the increase in the number of dynamic MT ends, we evaluated the consequences of exposing WT and TTR KO DRG neurons to the HDAC6 inhibitor ACY-738, which increases α-tubulin acetylation levels. To this end, levels of acetylated α-tubulin were evaluated in DIV4 DRG neurons isolated from adult WT and TTR KO mice by immunofluorescence. First, we confirmed that the neurite shafts of TTR KO neurons had decreased levels of acetylated α-tubulin when compared to WT controls (Figures 4A,B). More importantly, while addition of ACY-738 at this concentration did not affect WT neurons (Benoy et al., 2017), it normalized α-tubulin acetylation levels in the neurite shafts of TTR KO neurons (Figures 4A,B). Next, we analyzed whether the increase in α-tubulin acetylation promoted by ACY-738 in TTR KO axons had an impact on the number of dynamic MTs by measuring EB3 comet density in WT-Thy1-EB3-YFP and TTR KO-Thy1-EB3-YFP neurons. Indeed, EB3 live imaging confirmed an increase in comet density in TTR KO neurite shafts when compared to WT controls (Figures 4C,D), similarly to what was observed *ex vivo* (Figure 2F). However, this effect was completely reverted by ACY-738 (Figures 4C,D), strongly supporting that regulation of MT dynamicity by TTR is mediated by its modulation of α-tubulin acetylation levels. To clearly demonstrate that the observed phenotypes are TTR specific, we added TTR to DRG KO neurons and observed a complete rescue of both α-tubulin acetylation levels and EB3 comet density (Figures 4E,F).

To decipher how TTR modulates α-tubulin acetylation, we measured the levels of the tubulin acetylase ATAT1 and the deacetylase HDAC6 by Western blot analysis of protein extracts from WT and TTR KO DIV4 DRG neurons. We found that the decreased acetylated α-tubulin levels in TTR KO neurons correlated with decreased ATAT1 expression without an effect on HDAC6 (Figures 4G,H) suggesting that TTR modulates α-tubulin acetylation by regulating ATAT1 levels.

Finally, we tested whether TTR increases neurite outgrowth by interfering with α-tubulin acetylation, and assessed the effect of ACY-738 on the impaired neurite outgrowth of TTR KO neurons. Strikingly, while addition of ACY-738 did not affect neurite outgrowth of WT neurons, it rescued the ability of TTR KO DRG neurons to extend neurites *in vitro* (Figures 4I,J). These data demonstrate that TTR regulation of α-tubulin acetylation is required for its effect on axon growth. ACY-738 presents high specificity for HDAC6, although the inhibitor was also shown to inhibit other deacetylases (Benoy et al., 2017). As such, we cannot exclude that the inhibitor might be acting on other acetylases and/or affecting gene expression. Nevertheless, ACY-738 rescued α-tubulin acetylation levels, mitochondrial defects in DRG neurons, and motor and sensory axonal deficits, in a mouse model of the peripheral axonopathy Charcot–Marie–Tooth (Benoy et al., 2017).

Overall, our results reveal a novel activity of TTR in the regulation of the dynamics of axonal MTs as a result of modulation of acetylated α-tubulin levels, and demonstrate that this activity underlies the TTR-mediated promotion of axonal outgrowth. Additionally, we observed a decrease in ATAT1 levels in TTR KO neurons, which might suggest that TTR regulates the expression of the acetylase. Nevertheless, we cannot exclude that TTR-mediated regulation of acetylated α-tubulin might further occur *via* direct or indirect regulation of ATAT1/HDAC6 localization or activity. The resultant TTR modulation of α-tubulin acetylation and its impact on the ability of the protein to promote axonal growth is supported by the literature, as spatial regulation of α-tubulin acetylation and MT stability was shown to be important for axonal regeneration (Erturk et al., 2007; Rivieccio et al., 2009; Cho and Cavalli, 2012).

The observation of TTR modulation of acetylated α-tubulin is particularly interesting given that TTR KO mice display a sensorimotor impairment that starts at 6 months of age (Fleming et al., 2007). In mouse peripheral sensory neurons, acetylated α-tubulin is enriched in submembranous bands in the soma rather than in the cytoplasmic MT network (Morley et al., 2016), and this somatic enrichment is thought to tune the mechanical properties of the membrane. Indeed, neurons deprived of acetylated α-tubulin are less elastic and require more force to trigger the mechanosensitive channels, a property corroborated by the importance of acetylated α-tubulin in maintaining touch sensitivity in mechanosensory neurons (Morley et al., 2016; Yan et al., 2018). Based on this collective evidence, our observations strongly support that sensorimotor defects in TTR KO mice might be related to dysregulation of α-tubulin acetylation. TTR modulation of MTs might also underlie axonal transport impairment reported in TTR KO mice (Fleming et al., 2009). Importantly, loss of TTR function on MTs and axonal transport may occur in ATTR-PN contributing to sensory neuropathy, a topic that should be a subject for further investigation.

## Data Availability Statement

The raw data supporting the conclusions of this article will be made available by the authors, without undue reservation.

## Ethics Statement

The animal study was reviewed and approved by the protocols described in this work have been approved by the IBMC/i3S Ethical Committee and by the Portuguese Veterinarian Board.

## Author Contributions

MAL coordinated the research. JE, JM, and MAL conceived and analyzed the experiments. JE, JM, NM, and MEP performed the experiments. TM and MMS provided the critical tools and revised the manuscript. JE, FB, and MAL wrote the manuscript. All authors read and approved the final manuscript.

## Conflict of Interest

The authors declare that the research was conducted in the absence of any commercial or financial relationships that could be construed as a potential conflict of interest.

## Publisher’s Note

All claims expressed in this article are solely those of the authors and do not necessarily represent those of their affiliated organizations, or those of the publisher, the editors and the reviewers. Any product that may be evaluated in this article, or claim that may be made by its manufacturer, is not guaranteed or endorsed by the publisher.
