# Supplementary material for: Transthyretin Promotes Axon Growth via Regulation of Microtubule Dynamics and Tubulin Acetylation
Source: Front Cell Dev Biol. 2021 Nov 8;9:747699. doi: 10.3389/fcell.2021.747699 (PMC8606651; doi:10.3389/fcell.2021.747699)
Supplement: Supplementary file 7 [file Data_Sheet_1.PDF]

Supplementary Material

Supplementary Figure 1

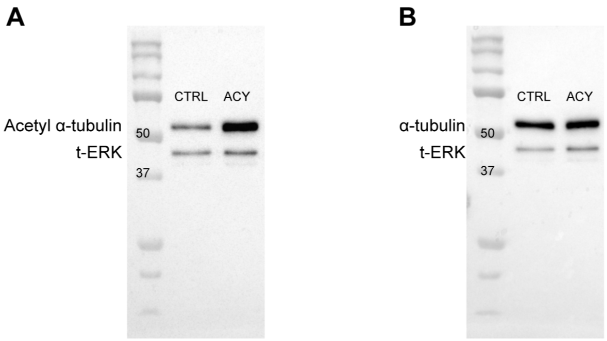

Supplementary Figure 1. Validation of anti-acetylated α-tubulin antibody. Representative western blots showing acetyl α-tubulin, α-tubulin and ERK levels in protein extracts from DIV4 DRG neurons untreated (CTRL) or treated with 2.5 μM Acy-738 (ACY).

Full length blots Figure 3

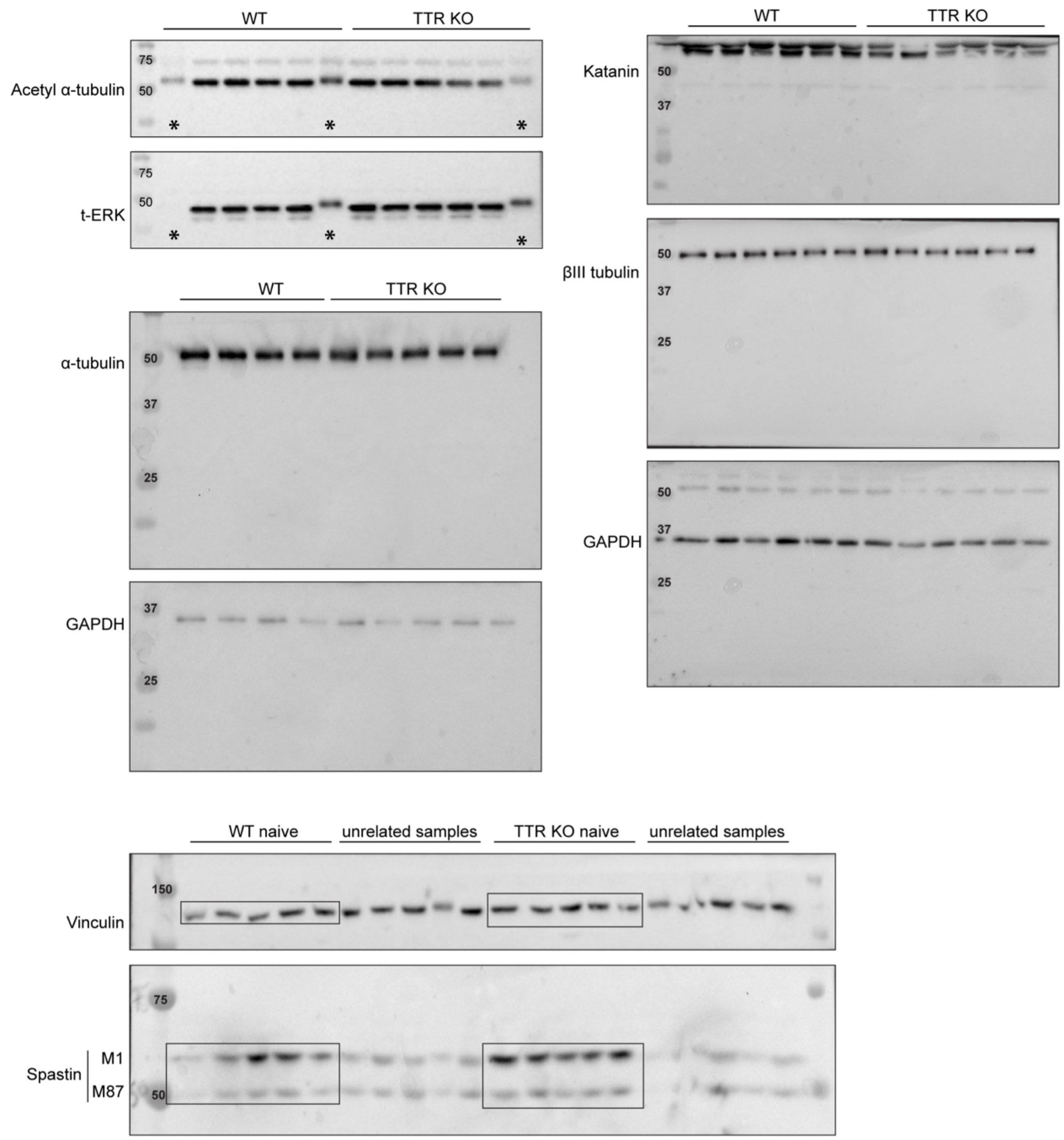

Bands presenting an altered running pattern (*) and samples unrelated to the present work were not quantified. Chemiluminescence images were merged with the ones showing molecular weight markers.

Full length blots Figure 4

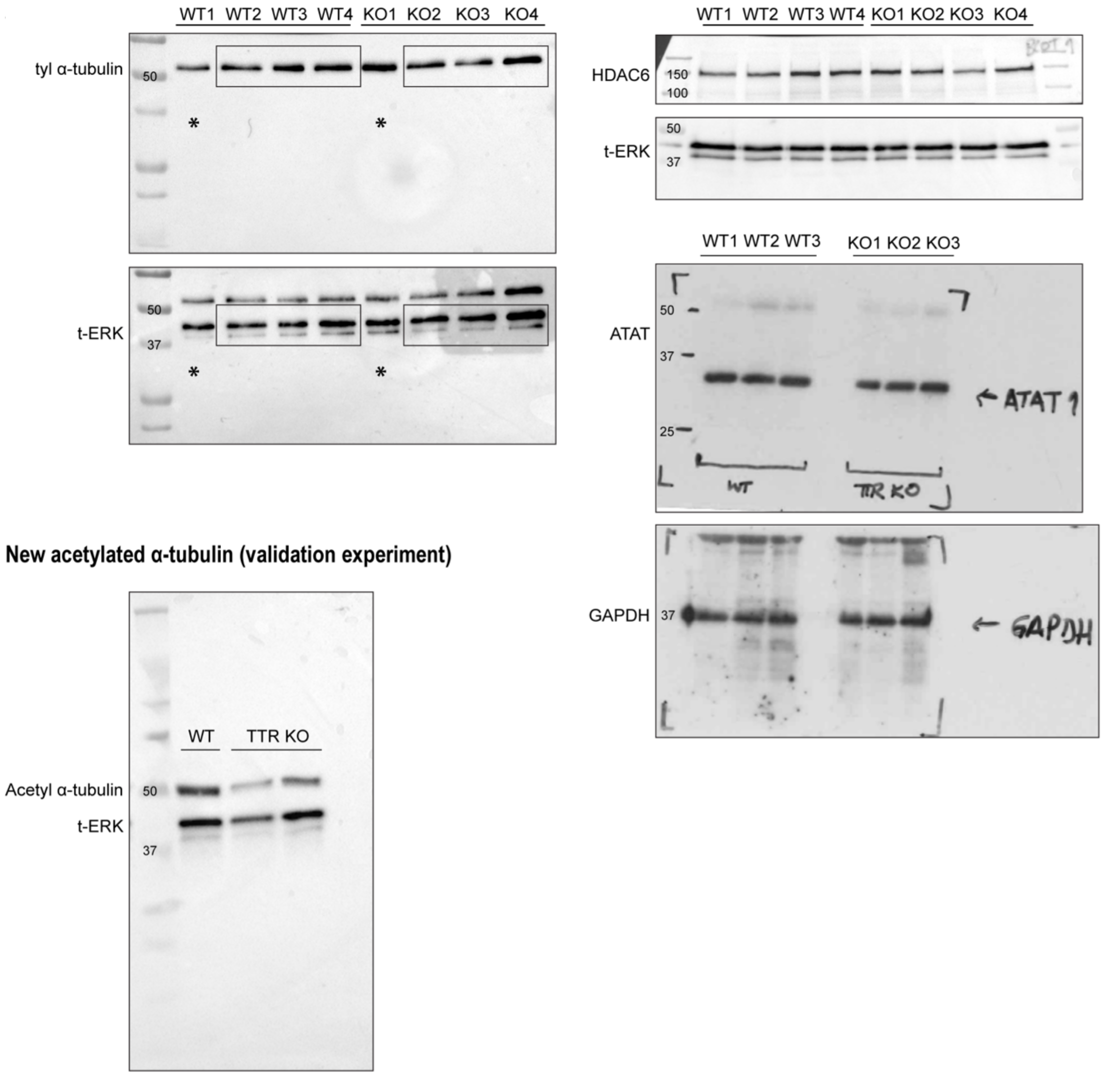

*Outlier experiment, as determined by Graph Pad analysis, was not quantified. An additional experiment was performed for validation. Chemiluminescence images were merged with the ones showing molecular weight markers.
